# Dehydration Influences Mood and Cognition: A Plausible Hypothesis?

**DOI:** 10.3390/nu3050555

**Published:** 2011-05-10

**Authors:** David Benton

**Affiliations:** Department of Psychology, University of Wales Swansea, Swansea SA2 8PP, Wales, UK; Email: d.benton@swansea.ac.uk; Tel.: +44-1792-295607; Fax: +44-1792-295679

**Keywords:** children, cognition, dehydration, elderly, hydration, mood

## Abstract

The hypothesis was considered that a low fluid intake disrupts cognition and mood. Most research has been carried out on young fit adults, who typically have exercised, often in heat. The results of these studies are inconsistent, preventing any conclusion. Even if the findings had been consistent, confounding variables such as fatigue and increased temperature make it unwise to extrapolate these findings. Thus in young adults there is little evidence that under normal living conditions dehydration disrupts cognition, although this may simply reflect a lack of relevant evidence. There remains the possibility that particular populations are at high risk of dehydration. It is known that renal function declines in many older individuals and thirst mechanisms become less effective. Although there are a few reports that more dehydrated older adults perform cognitive tasks less well, the body of information is limited and there have been little attempt to improve functioning by increasing hydration status. Although children are another potentially vulnerable group that have also been subject to little study, they are the group that has produced the only consistent findings in this area. Four intervention studies have found improved performance in children aged 7 to 9 years. In these studies children, eating and drinking as normal, have been tested on occasions when they have and not have consumed a drink. After a drink both memory and attention have been found to be improved.

## 1. Introduction

We are frequently told that we consume too little fluid with the potential for adverse consequences of both a physical and psychological nature. Amongst the earlier symptoms of dehydration are headaches, feeling tired and light-headed. Over recent years a lucrative market has been created by selling bottled water. In western societies water bottles are often carried; for example they may be seen attached to rucksacks or placed on a desk. To what extent does this phenomenon meet a basic physiological need rather than being a reflection of a successful marketing strategy? Even if increased water consumptions improves aspects of basic physiology, or decreases the risk of disease, is there any evidence that the level of dehydration experienced by those leading a typical life style, in a temperate climate, disrupts mood and cognitive functioning? Certainly there are many popular suggestions that mild dehydration is a common phenomenon with statements such that water is “truly a wonder drug” and that eight glasses a day will improve your energy, increase your mental and physical performance and help you lose weight [1].

About sixty percent of the body is made up of water where it is involved in a wide range of basic functions; in fact it is a major constituent of every cell. In the blood it transports oxygen, nutrients and waste products; the mucus membranes of the lungs need to be moist; its loss as perspiration cools the body; it is required for the digestion of food. Without any intake of fluid death can occur after three to five days, depending on environmental conditions such as temperature and activity levels. Yet we loose water when we breath, perspire, urinate or defecate and thus need to replenish lost fluid. On average an adult produces about one and a half litres of urine a day with another litre being lost by breathing, perspiration and bowel movements. Although food offers about a fifth of the total intake of fluid there remains a need to consume about two litres of water, or other beverages, to make good the loss. Particularly in the United States this message has been summarized in the “8 × 8 rule”: That is eight times a day we should drink an eight ounce glass of water (about 1.9 L). There are higher estimates; Kleiner [2] stated that “to be well hydrated, the average sedentary adult man must consume at least 2900 mL fluid per day, and the average sedentary adult woman at least 2200 mL”. The Institute of Medicine of the National Academies [3] suggested that an adequate intake for women was approximately 2.7 L of water a day, with the figure for men being 3.7 L. The figure for children from one to three years was 1.3 L and from 4 to 8 years 1.7 L, whereas boys from 9 to 13 years should consume 2.4 L and girls of this age 2.1 L. About 80% of these values come from the drinking water and beverages with the other 20% coming from food. The European Food Standards Agency [4] recommended an intake of “1600 mL/day for boys and girls 4-8 years of age; 2100 mL/day for boys 9-13 years of age; 1900 mL for girls 9-13 years of age”. Questions arise as to life style? These recommendations are for those leading a sedentary life so the figures need to be increased if you are particularly active or the weather is hot. It is apparent that such recommendations vary considerably illustrating that this is an area of some uncertainty. 

Children in particular are said to be at risk of dehydration as they are often dependent upon others for the provision of fluid, they are more active and they have a greater surface-to-mass ratio than adults. In the United Kingdom the Royal College of Paediatrics and Child Health and a charity dealing with continence in childhood surveyed drinking facilities in schools. The resulting report claimed that access to water was often unsatisfactory. There resulted the “Water is Cool in School” campaign [5] that aimed to improve access to fresh drinking water. It was claimed that dehydration contributes to both short and long-term health problems and that drinking more water improves the ability to learn as “when we are thirsty, mental performance deteriorates by 10%”. The basis for this number is unclear, particularly as the multi-faceted nature of cognition means that it cannot meaningfully be summarized as a single number.

## 2. Osmolality

Plasma volume and osmolality need to be within a limited range, a situation that is maintained by hormonal mechanisms and behaviour that leads to fluid ingestion and the conservation of water and sodium. Osmolarity is a measure of solute concentration, that is the number of osmoles of solute per litre of solution. Molality is the number of osmoles of solute in a kilogram of solvent. Osmolality is the more commonly quoted measure. Homeostatic mechanisms try to maintain osmolality within a prescribed range.

There are both intracellular and extracellular (blood) mechanisms. An increase in osmolality will result in water leaving cells and entering the blood. Such dehydration will be monitored by osmoreceptors in the brain that initiate drinking behaviour and the release of vasopressin from the pituitary gland. The hormone vasopressin (arginine vasopressin, anti-diuretic hormone; ADH) plays an essential role in this process as without this hormone little water is reabsorbed and dilute urine is excreted. Vasopressin causes the kidney to produce a lower volume of urine and hence retain water. Extracellular dehydration (hypovolaemia or lowered blood volume) stimulates receptors that send signals to the brain and hence starts drinking behaviour and the release of vasopressin. The kidneys by controlling the excretion of sodium and water keep the osmolality of the extracellular fluid within narrow limits. An increase in osmolality of only one to two percent increases the desire to drink and induces maximal water conservation by the kidney. The kidneys can concentrate urine and thus conserve water, or alternatively they can get rid of excess water by producing dilute urine. 

Receptors in the hypothalamus respond to any increase in plasma osmolality by stimulating the secretion of vasopressin. In addition, in the heart stretch receptors are activated when there is an increased volume of blood returning to the heart. In this way vasopressin secretion is inhibited and excess fluid volume is excreted. Also stretch receptors in the carotid artery and the aorta are stimulated if blood pressure decreases. Vasopressin is released causing the volume of liquid in the blood to rise and in this way blood pressure is increased [6].

In addition to controlling the level of Total Body Water the amount of solute is also tightly monitored as variations in osmolality will cause cells to swell or shrink with consequences for both structure and functioning. Critically a balance must be maintained between the intake and excretion of sodium, the main solute in extracellular fluid. It is obvious that sodium and water levels must be integrated as changes in the volume of water will influence the concentration of sodium. As you become dehydrated proportionately more water is lost than sodium, increasing osmolality, so that water should be conserved but not sodium.

Although vasopressin lowers osmolality by encouraging the kidney to reabsorb water, there are also mechanisms that prevent osmolality falling to undesirably low levels. Aldosterone, a hormone created by the adrenal cortex, influences the reabsorption of sodium by the kidney. The adrenal cortex monitors plasma osmolarity and when it reaches above normal levels the release of aldosterone is inhibited causing less sodium to be reabsorbed. In addition the kidneys respond to low blood pressure by producing renin that initiates a metabolic sequence that results in the production of angiotensin II, that in turn causes the adrenal cortex to release aldosterone. 

The importance of keeping osmolality within a narrow range, and the sophisticated physiological mechanisms that have developed to achieve this end, leads to the hypothesis that short-term and minor differences in fluid intake are unlikely to influence osmolality and hence will have neither significant physiological nor psychological consequences. Rather than seeing dilute urine as desirable and moderately yellow urine as a sign of dehydration, the former could be viewed as having consumed too much liquid and the latter that normal mechanisms have kicked in to prevent dehydration. A priori it seems improbable that evolution has resulted in a creature where the maintenance of appropriate osmolality is essential, yet in those following sedentary lives in temperate climates there are frequent deviations from this status with negative consequences. Is it really the case that many people consume too little water?

Three phenomena need to be distinguished. Hypotonic or hyponatremic dehydration results from a loss of electrolytes, in particular sodium; hypertonic or hypernatremic dehydration reflects a loss of water; isotonic or isonatremic dehydration is characterized by a loss of both water and electrolytes. In hospital hyponatremia is the most common electrolyte disorder occurring in about 5% of inpatients. However, overall by far the most common type is isotonic dehydration which effectively equates with hypovolemia, a reduced volume of blood plasma. The type of dehydration is, however, important. A characteristic of hypotonic dehydration is the movement of intravascular water into extravascular space that further reduces the intravascular volume for a given decline in Total Body Water. A hypotonic state can result in seizures, whereas rapid rehydration after a hypertonic state can produce osmotic cerebral oedema.

The control of serum osmolality and sodium concentration is, however, under normal circumstances tightly controlled. If the osmolality of extra-cellular fluid increases by 2%, osmoreceptors in the hypothalamus stimulate thirst and release vasopressin with consequent water conservation. Further water loss will decreases blood flow to the kidneys stimulating the production of renin, angiotensin and aldosterone, thus preventing a loss of sodium [6,7]. For an adult, urine has a typical osmolality of 1200 mosmol/L (although this can range from 40 to 1400 mosmol/L). Infants urine is more dilute, with a typical value of about 700 mosmol/L, as they have a lower capacity to concentrate urine [8]. 

Valtin [9] considered the suggestion that one cause of dehydration was that the sensation of thirst occurred only after an individual was already dehydrated. He stated that in fact thirst develops when you are only slightly dehydrated, a loss of 0.8-2% loss of body weight due to water loss. At this point the average plasma osmolality is 294 mosmol/kg, when the average plasma osmolality when thirst is not stimulated is 287 mosmol/kg and the normal range is 280-296 mosmol/kg. Critically the threshold for the release of vasopressin is 284.7 mosmol/kg, a point lower than is needed to generate thirst and hence the desire to drink. It seems that physiological mechanisms kick in at a relatively early point to prevent the development of dehydration. 

Given the efficiency of these homeostatic mechanisms, a priori, it would seem unlikely that failing to drink for relatively short periods in a temperature climate would disrupt bodily functioning. The question arises as to whether there is evidence that failing to drink for a few hours results in a degree of minor dehydration associated with adverse psychological consequences? The Institute of Medicine of the National Academies [3] concluded that “the vast majority of healthy people adequately meet their daily hydration needs by letting thirst be their guide”. Kleiner [2], however, noted that nutrition surveys suggest that a section of the population was chronically mildly dehydrated as their fluid intake was considerably less than the levels recommended. Such a conclusion is, however, valid only if the recommended levels of intake are accurate.

## 3. Assessing Dehydration

Basic to the study of dehydration is the measurement of hydration status, a problem for which there is no easy solution given the intricacies of the underlying mechanisms. Water is found in various compartments: Intracellular fluid accounts for about 55% of Total Body Water, interstitial fluid for about 20% and intravascular fluid for about 7.5%. It is inevitable that no single measure can adequately reflect a dynamic and complex mechanism. 

Armstrong [10] reviewed thirteen methods of assessing hydration that included assessing the whole-body, blood, urine and the taking of sensory measures. He concluded that “A single gold standard … is not possible for all hydration assessment requirements”. For example he considered the claim that a Total Body Water value provided the “gold standard”: That is the use of isotope dilution and neutron activation analysis. The method, however, requires laboratory facilitates with highly controlled conditions such as maintaining the same posture, diet and general environmental conditions. The isotope dilution measurements require three to five hours to come to equilibrium, a process that will be influenced by general activity. Clearly repeated measurement is not a possibility and the approach cannot deal with short-term changes. 

Similarly plasma osmolality, although widely used, is subject to the criticism that it fails to produce valid measures in all settings. For example there is a report that osmolality increased over three days of water restriction, although on the first day of over-hydration the measure did not change and did not parallel the increase in body weight that resulted from water consumption [11]. Armstrong [10] concluded that plasma osmolality is particularly misleading when Total Body Water, fluid intake and fluid loss are fluctuating. 

When assessing hydration outside the laboratory a range of additional practicalities arise. It is simply not feasible to use many laboratory methods when assessing the association between short-term changes in psychological functioning and repeated measures of hydration status. Anderson [10] concluded that changes in body weight offer the “simplest and most accurate index of hydration status in real time, when serial measurements are made in close proximity”. Such an approach, however, requires the use of weighing scales of a greater accuracy and sophistication than would typically be used to measure body weight. Electronic scales that can average perhaps fifty assessments of weight over a five second period, and in this way get around the effect of body movement on the measure, allow changes of a few grams in body weight to be established. The use of such a technique has the sensitivity to monitor, over short periods, even minor losses of fluid associated with breathing and perspiration. Such an approach offers a practical means of repeatedly assessing relatively small changes in hydration, over a few hours in real world settings, allowing the hypothesis that minor changes can have adverse psychological consequences to be addressed. Dehydration has been defined as a loss of more than 2% of body weight and mild dehydration as a loss of between 1% and 2% [2].

## 4. Is There Evidence of Widespread Dehydration?

With an orthodox view on any topic the basic evidence on which it is founded may be infrequently considered and can even have become largely forgotten. Valtin [9] explored the origin of the recommendation to drink “8 × 8” and was unable to conclusively establish its origin. The best he could do was to find a recommendation along these lines in 1974, although the suggestion was not argued or based on quoted evidence. 

Valtin [9] considered the scientific study of the influence of the volume of water consumed on physiology and disease. A common finding in surveys of fluid intake is that large sections of populations do not consume the amount recommended although there is no evidence of health problems. He reviewed the influence of drinking more water on a range of medical parameters and concluded that “no scientific studies were found in support of the suggestion that we should drink eight glasses of water a day”, at least in healthy individuals in temperate climates living a sedentary lifestyle. He suggested that as the release of vasopressin occurs before thirst is experienced, necessary changes in water balance are met by changes in urine flow. The mechanisms are so quick and accurate that “it is hard to imagine that evolutionary development left us with a chronic water deficit that has to be compensated by forcing fluid intake” [9]. 

It should be remembered that the conclusions of Valtin [9] are limited to the healthy and sedentary living in a temperate climate and that enhanced fluid intake has a place in the treatment of some diseases. Meinders and Meinders [12] came to similar conclusions. They commented that under normal circumstance the minimal urine output is about 500 mL/day. They suggested that the consumption of more than 500 mL/day is adequate and that the recommended consumption of three litres a day is more than adequate. They commented that there was no convincing health benefits associated with a higher fluid intake with the possible exception of preventing kidney stones.

## 5. Hydration Status and Cognition

There has been surprisingly little study of the influence of dehydration on cognition and where it has been carried out it has been mainly with young rather than older adults or children. With adults, those tested in extreme situations such as in high temperatures or taking part in prolonged exercise or in military settings need to be distinguished from those following a sedentary life in a temperate climate.

In fact some studies have used extreme conditions to generate dehydration, for example undertaking a military exercise for 53 h in hot conditions, during which they slept for an average of three hours [13]. They lost on average 4.1 kg of weight, the majority of which reflected the loss of body fluid. Unsurprisingly mood and the performance of a range of cognitive functions, including vigilance, reaction times, attention, memory and reasoning was impaired. As cold exposure can induce dehydration, in a similar study of the military [14] the effect of dehydration was considered. Three hours spent at 4 °C was followed by cycling for one hour with or without fluid replacement, that in the latter instance reduced body weight by three percent. However, dehydration “did not alter any cognitive, psychomotor, or self-report parameter”. Such studies preclude the distinguishing the effect of dehydration from a range of other stressors.

### 5.1. Exercise Induced Dehydration

Although there are frequent anecdotal reports from athletes that exercise-induced dehydration adversely effects cognition, it is impossible for the individual to distinguish one aspect of a complex situation from another, and controlled studies have not generally supported such a role for hydration.

In adults, when a reduction in body weight of up to 4% was produced by step-up exercises at 45 °C, attention, memory and arithmetic ability were compromised, with significantly reduced performance being associated with a more than 2% reduction in body weight [15]. A similar study also used heat and exercise, running on a treadmill for two hours, to induce dehydration that resulted in a weight loss of up to 2.8%. Although short-term memory [16,17], decision making [16,17], psychomotor control [17] and perceptual discrimination were poorer, simple reaction times and long-term memory were unaffected. It was suggested that more demanding tasks might be more susceptible to dehydration. Importantly subsequent fluid ingestion did not reverse these decrements [16,17].

Although step-ups at 45 °C followed by cycling reduced body weight by 1, 2, or 3%, the performance on a symbol substitution task was not influenced, although a loss of more than 2% of body weight reduced working memory and psychomotor functioning [18].

However, not all studies have found such decrements in performance. Cycling for three hours without fluid ingestion resulted in a 4.1% loss of body weight that was only 2.2% when fluid was provided. The performance on a critical flicker fusion test and a map recognition task was not influenced by fluid consumption [19]. Similarly when cycling at 31 °C the consumption of fluid equivalent to 0, 50 or 100% of fluid loss did not influence reaction times: That is they were not compromised by dehydration [20].

When college athletes trained without the opportunity to drink water they typically lost between 1% and 2% of their body weight during a training session. When they had trained without rather than with the opportunity to drink, their mood was rated more negatively, although memory as assessed using Digit Span was better. Vigilance was, however, better on the occasions when water had been drunk. There was, however, an interaction between hydration status and the sex of the athlete when reaction times were assessed [21]. However, playing football that produced mild/moderate dehydration associated with up to 2.5% loss in body weight had no effect on cognitive functioning. In addition the change in osmolality did not predict changes in cognitive functioning [22]. Similarly a study examined those who played football after 45 min of pedalling a bicycle, who on different occasions did or did not consume fluid. There resulted a 1.5-2% loss of weight. The post-match performance of a sports physical fitness test was impaired when there had been no fluid intake. However, the performance of a test of mental concentration was similar irrespective of whether fluid had been consumed. This lack of change resulted even though plasma osmolality increased from 290 to 305 mosmol/kg H_2_O when not drinking, whereas when fluid was consumed it only increased from 290 to 293 mosmol/kg H_2_O. Urine osmolality increased from 360 to 620 mosmol/kg H_2_O when not drinking and declined from 530 to 520 mosmol/kg H_2_O when water was drunk [23].

Playing basket-ball for up to two hours without the consumption of fluid reduced body weight by 1.3% after 15 min, 2.3% after 60 min and 3.7% after two hours; figures that compared with a loss of only 0.7% after two hours when fluid was consumed. Choice-response times and short-term memory was poorer following exercise but dehydration did not influence the performance of these cognitive tests [24]. In contrast when basketball players were asked to walk at 40 °C and then play a simulated game when euhydrated or 1-4% dehydrated, a computerized measure of vigilance was impaired by dehydration [25]. 

A study that used a long-term less strenuous form of exercse introduced an important dimension, the age of the subjects. Individuals who undertook 10 days of strenuous hill walking (daily walking distances were 21 km (10-35 km) with an ascent of 1160 m (800-2540 m)). They also completed tests of muscular strength, cognitive processing time and flexibility. A group with an average age of 24 years remained hydrated, whereas a second group with an average age of 56 years groups became dehydrated, reflecting differs in the decision to consume liquid,. In fact in the older group the urine osmolality increased from a baseline value of 600 mosmol/L to 750 mosmol/L at six days and to 1000 mosmol/L at day 11. The increase in urine osmolality in this older group correlated with the height to which they could jump and a decline in choice reaction times [26].

### 5.2. Evaluation of Exercise Induced Dehydration

There is considerable interesting in those studying sporting performance in the need to maintain hydration. When Shirreffs [27] reviewed the topic she concluded that the performance of endurance exercise in hot conditions (31-32 °C) was impaired by a dehydration induced loss of 2% body weight. However, at 20-21 °C the loss of 2% of body weight was inconsequential and in a cold environment tolerable. She concluded that the evidence concerning the skill associated with sport and for mental performance was inconclusive. 

In fact the evidence concerning mental performance after exercise induced dehydration is mostly negative and where positive difficult to interpret. The correlational nature of the data creates a problem. It has been suggested that measures of dehydration after exercise may be only a marker for outcomes resulting from enhanced metabolic activity. The complexity of the mechanisms that control metabolism make it unlikely that a single physiological system, such as hydration, will explain the entire picture [28]. It is similarly unclear whether any decrement in cognitive functioning after exercise reflects fatigue, heat stress or dehydration. In particular the use of a hot environment causes problems as an increased core body temperature is known to disrupt cognitive functioning. Such studies have almost without exception used young, trained very fit individuals such that the data should not be uncritically generalized. 

There has been a suggestion on several occasions that the loss of 2% of body weight is critical, however, although a loss of more than 2% had been reported to disrupt some measures of cognitive functioning [15,16,17,18] as frequently it has not been disruptive [19,22,24]. That the subsequent provision of fluid has not reversed these decrements [16,17] questions whether hydration is the underlying mechanism. Some evidence that hydration may be the mechanism comes from studies where the fluid was or was not provided while the body was subject to dehydrating conditions. Although there are reports that the provision of fluid was helpful [21,25] this has not always been the case [23].

A study considered possible changes in brain volume after exercise and heat-induced dehydration that resulted in a 2.9% loss of body mass [29]. The volume of the brain did not change suggesting that mechanisms are in place to preserve its structure, although there were reductions in the volume of ventricular and cerebrospinal fluid. There is, however, a report using serum S100beta concentrations, as a marker of Blood Brain Barrier permeability, that exercise induced dehydration increased permeability, a response that was reduced by water consumption [30].

It is clear that even with the relatively large losses of fluid induced by heat and exercise the evidence that dehydration is cognitively disruptive is limited and inconsistent. Even if it is the case that a loss of 2% of body weight is critical, how typical is it of individuals going about their everyday life? 

### 5.3. Fluid Deprivation

Studies where the degree of hydration was manipulated by the differential provision of fluid are easier to interpret and more typical of everyday life. However, even in such studies the interpretation is not straightforward; for example if the consumption of additional fluid reduces a rise in body temperature then hydration as such may not be the critical variable.

A study specifically examined the proposition that cognition is adversely influenced by moderate dehydration [31]. Young adults were examined twice, after normal water consumption or deprivation for 24 h. A 2.6% decrease in body weight resulted from being deprived of water for this period. Neither the performance of a battery of cognitive tasks nor event-related potentials were influenced by dehydration. However, self-ratings of feeling tired and less alert resulted and there was greater perceived effort expended when performing the cognitive tests. There were gender differences in some tests that relied on reaction times; responses improved in males when dehydrated but got worse in females. They concluded that “cognitive-motor function is preserved during water deprivation in young humans up to a moderate dehydration level of 2.6% of body weight”. In this study serum osmolality increased from 290 mosmol/kg H_2_O at baseline to 296 mosmol/kg H_2_O after 24 h of water deprivation. Urine osmolality increased from a baseline value of 691 mosmol/kg H_2_O to 1004 mosmol/kg H_2_O after 24 h of water deprivation.

Similarly subjects were required not to consume any fluids and to eat foods with a low water content for 37 h [32]. Body mass fell by 1% after 13 h, 1.8% after 24 h and 2.7% after 37 h. At baseline plasma osmolality was 281 mosmol/kg H_2_O but increased to 287 mosmol/kg H_2_O after 13 h and 291 mosmol/kg H_2_O after 24 and 37 h. These figures put any likely daily variation in hydration status into perspective. It is unlikely that many, if any, do not drink for 13 h, yet plasma osmolality was within the normal range after this period. Although it has been suggested that we need to lose 2% of body weight for adverse psychological responses to occur, there was only a 1% decline in body weight after 13 h without any liquid. Although the subjects rated themselves as less alert but not less tired after 13 h without fluid, such a degree of deprivation is unlikely to occur under normal living conditions. 

### 5.4. Dehydration in Older Adults

The few studies of free living individuals have considered older adults. In such a sample poorer hydration was associated with poorer attention and memory [33], although there was no attempt to demonstrate a causal relationship by examining the response to drinking water. Similarly dehydration was examined using bioelectrical impedance in a sample of females, mean age 60 years, who were living in the community [34]. Total Body Water by weight was found to be related to measures of memory, although when diastolic blood pressure was taken into account the association between hydration and cognition attenuated. They concluded that when considering the cognitive functioning of older adults it was important to take into account both hydration and blood pressure. Similarly 10 days of strenuous hill walking resulted in dehydration in group with an average age of 56 years, but not a younger sample. The increase in urine osmolality in the older group correlated with a decline in choice reaction times [26].

Renneboog and Musch [35] examined the frequency with which individuals fell over and hurt themselves, in a sample with an average age of 72 years with chronic hyponatremia without any overt symptoms (mean serum sodium concentration 126 ± 5 mEq/L): 21.3% were admitted into hospital after falling, compared with only 5.3% of a control group. The speed of responding to the tests of attention and the number of errors were poorer in those with hyponatremia.

These are potentially important observations as the ability to maintain hydration may decline with age, for example the sensation of thirst progressively declines [36]. However, the data are limited and there has been no attempt to demonstrate a causal role for dehydration by varying the provision of fluid.

### 5.5. Increasing Fluid Intake

One of the few intervention studies in this area found that when thirst was high the consumption of water had a positive influence on sustained attention; whereas when subjects were not thirsty drinking water was detrimental [37]. In contrast, irrespective of initial thirst, ratings of alertness increased after drinking water. A later study considered those who had not eaten or drunk from mid-night the previous evening [38]. The following day the cognition of those who drank nothing was similar to those who consumed 150 mL of water although self-ratings of arousal increased. 

There has also been a suggestion that there is an interaction between thirst and the reaction to other aspects of nutrition. After rating their thirst, subjects drank either a glucose drink or a placebo. In a memory test those who were less thirsty performed better following the consumption of glucose rather than a placebo. In contrast the memory of those who were initially thirsty was worse after glucose rather than the placebo. The response to glucose depended on the participants’ initial thirst [39]. However, unlike previous reports [37,38] initial thirst did not influence subsequent ratings of alertness. 

This is a topic that has been little researched and the findings to date have been inconsistent. There are, however, a series of significant findings that may benefit from systematic study.

### 5.6. Hydration Status in Children

The study of hydration in children is even more limited, particularly of those living in temperate climates. However, in an Israeli school, in the desert where it was 35 °C degrees outside and 30 °C indoors, dehydration was indicated by urine osmolarity [40]: values <500 mosmol/kg H_2_O represented adequate hydration. However, the mean urine osmolality was 862 ± 211 mosmol/kg H_2_O and 67.5% had values above 800 mosmol/kg H_2_O and in 25% the values were greater than 1000 mosmol/kg H_2_O. In the morning the scores on five cognitive tests did not differ in those who were and were not dehydrated. However, in this sample of ten year olds, during the afternoon the performance of one out of five tests was significantly better in those with better hydration. The authors noted the possible confounding effect of heat stress. 

In 2006 a review of the association between the hydration of children and cognition was unable to find a single intervention study without which causality cannot be demonstrated [41]. In fact the correlational study [40] was the only one at this time that had examined the association between hydration and cognition in children. More recently, in Sicily, the hydration status was estimated of 168 school children who were in classes that either did or did not have additional water provided [42]. The ambient temperature was about 25 °C. A significant relationship was found between urinary osmolarity and digit span, a measure of working memory. Children who were more dehydrated tended to have poorer memories. Perhaps because the control group could drink water ad libitum, and some of them drank quite a lot, there was no significant difference in memory when additional water was provided, although most of the children offered additional water did not drink all that was provided. 

Even if a significant association had been demonstrated between osmolality and cognition the finding would need to be interpreted with caution. In a sample of German children those who were more hydrated not only drank more water but also consumed more water-supplying foods and less energy from fat [43]. This finding raises the possibility that the hydration status of children is a marker for the overall quality of the diet. If so, it is not possible to establish whether any association between hydration and cognitive functioning reflects a lack of water, rather than generally poor nutrition. There is a need for intervention studies, as data based solely on physiological measures of hydration status are impossible to interpret.

#### 5.6.1. Intervention Studies in Children

Ultimately a correlation between a measure of hydration and an aspect of cognition can never demonstrate causality. To date studies have typically included obvious confounding variables such as a high ambient temperature, prolonged exercise or sitting in a sauna. This is not, however, an area where a double-blind trial can be run as people are clearly aware if they have or have not drunk recently. An intervention study does, however, offer the advantage of demonstrating that a deficit observed in those with a low fluid intake can be reversed by drinking. Although logically you cannot exclude a placebo effect, it is a necessary albeit not sufficient condition when trying to demonstrate an adverse reaction to dehydration. More recently there has been a series of intervention studies of children in which behaviour has been compared when water has and has not been consumed. Given the consistency of these findings they will be described in more detail.

#### 5.6.2. Benton and Burgess [44]

On two afternoons the cognitive functioning of forty children (mean of eight years and seven months) was assessed once after drinking water and on another day when no water had been consumed [44]. The minimum temperature of the class room was 20 °C at a time of the year when the external temperature was a maximum of 3-5 °C. The water was consumed at the beginning of the mid-afternoon break and testing took place 20-50 min afterwards. The children received a drink of 300 mL water that was always totally consumed. Depending on the school routine water was consumed at 14:00 in one school and at 14:30 in the second. No attempt was made to influence the normal pattern of eating and drinking. Lunch was eaten between 12:00 and 13:00 where a drink of 200 mL was provided. It was the policy of both schools to allow drinking: Both had water fountains near to the classrooms and water bottles were carried by some children. Thus the sample was one where there was ready access to liquid should the child choose to drink.

Memory was assessed by asking for the recall of a series of objects presented as a picture: It was significantly better on the day when water had been drunk. The ability to sustain attention was also measured by asking the child to respond to a light that followed an auditory warning after a delay of either three or twelve seconds. Again the ability to sustain attention was significantly better on the day when water had been drunk. 

#### 5.6.3. Edmonds and Burford [45]

Fifty-eight children aged 7-9 years were divided into two groups, one that received a drink of water and one that did not. In a particular class half of the children received water and half did not. Although the classroom temperature was not recorded the mean outdoor temperature was 5.6 °C and 9.9 °C on the days of testing. Bottles initially contained 250 mL of water and scales were used to weigh any water that remained. A mean of 212 mL was drunk. The time of day when testing took place is not reported but it began twenty minutes after drinking. 

Those who drank water performed a visual attention and a memory task better, although a visuo-motor tracking task was not affected by the consumption of water. They concluded that “even children in a state of mild dehydration, not induced by intentional water deprivation or by heat stress and living in a cold climate, can benefit from drinking more water and improve their cognitive performance”. 

#### 5.6.4. Edmonds and Jeffes [46]

Twenty-three children with a mean age of 7 years 3 months participated in the study. Eleven received water and 12 did not. On the day of testing the classroom temperature was 20 °C. Baseline testing began at approximately 09:30 and lasted twenty minutes. After another forty minutes half the children were given a 500 mL bottle of which on average 409 mL was consumed. The test session began 45 min after the drink. The group who drank water rated themselves as happier. Visual attention and visual search were better in those who had drunk, although visual memory and visuo-motor performance were not altered. 

#### 5.6.5. Benton and Davis [47]

Twenty-two children, aged nine years, were observed on six occasions a week apart. On three occasions they had drunk 200 mL of water at 14:30, and on three occasions water had not been drunk. On any one day half the children in a particular class received a drink and the other half did not. After fifteen minutes the children’s behaviour was observed for thirty minutes while working as individuals, writing or solving mathematical problems. The classrooms were maintained at a temperature of 24 ± 1 °C. On 58% of occasions the children had drunk both at breakfast and lunch and on thirteen percent of occasions three or more times. 

Using activity sampling a child was monitored on thirty occasions over a thirty minute period, precisely when an electronic bleep was heard via an ear-piece. The behaviour was assessed as being on task or off task (looking around; talking; fidgeting; out of seat). The data that resulted were the percentages of time that the children were on task in six five minute blocks. 

Table 1 illustrates the findings. On average the children spent 78.8% of their time on task after drinking water but significantly less time, only 53.0%, when water had not been consumed (F(1,21) = 88.82, *p* < 0.0001). The effect of drinking water persisted for the entire thirty minute period of observation. 

**Table 1:** The influence of consuming water on the percentage of time spent on task by school children.

Minutes of observation	Water	No water
1 to 5	85.4 ± 1.8	57.2 ± 3.8
6 to 10	82.1 ± 1.9	55.8 ± 3.6
11 to 15	79.1 ± 2.8	55.2 ± 3.0
16 to 20	79.4 ± 2.8	54.1 ± 3.5
21 to 25	73.9 ± 3.1	48.3 ± 3.7
26 to 30	72.7 ± 2.3	47.6 ± 3.2

The data are the average percentages of time spend on task with associated standard errors, when observed on three occasions after drinking water and three occasions when no water was consumed. The main effect of whether water had or had not been consumed reached statistical significance (F(1,21) = 88.82, *p* < 0.0001).

Arguably more time and effort was spent doing school work but alternatively after drinking water they might have been working more inefficiently, taking longer to finish their task. Both the observation of the children and previous use of this measure [48] suggested that the response to water consumption was positive and that the children were paying more attention to their work. The additional time on task occurred throughout the thirty minute period, demonstrating that those who had not drunk water were not initially working harder and finishing sooner. 

#### 5.6.6. Evaluation of Water Consumption and Children

Thus there are four studies that report that giving water to children improves some aspects of cognition. These results suggest that at this age even mild dehydration in a temperate climate can have negative consequences. A degree of dehydration that results from exercise or heat exposure was not necessary to observe these phenomena. On four occasions attention was better [44,45,46,47] whereas twice memory was improved [44,45], although on a third occasion it was not [46]. 

The consistency of these findings raised the obvious possibility that children between the ages of 7 and 9 years may suffer with dehydration to the extent that cognitive functioning is compromised. We await further studies that establish the aspects of cognition that are compromised, the age range of children that are affected, the effect of ambient temperature, the optimal fluid intake and the pattern of consumption, whether the recommended levels of intake are appropriate in this context? 

There have been previous suggestions that children fail to drink sufficiently. A British survey distinguished schools in terms of their drinking policy [49]: Those who prohibited water in the classroom; those with limited access where water was allowed in the class but not on the desk; those with free access to water with a bottle on the desk. Eighty one percent of the children in schools who prohibited the introduction of water into the class, and 80% of children who had limited access to water, drank less than the recommended amount. In contrast only 46.5% of those given free access to water drank less than recommended. Such findings are, however, difficult to interpret in the present context as the recommended levels of consumption are not based on psychological measures.

## 6. Discussion

Although there are repeated suggestions in the popular media that there is a widespread problem created by the consumption of low volumes of liquid [1], academic reviews have repeatedly failed to find supporting evidence [27,41,50]. When considering this topic three groups of subjects need to be distinguished depending on age; younger adults, older adults and children. 

Most research has been carried out on young fit adults, who typically have exercised, often in heat. The results of these studies are inconsistent such that no conclusion can be drawn. Although it is inevitable that with progressive dehydration at some point cognitive functioning will be disrupted, the impression gained is that a dehydration induced loss of body weight of 2-3% does not reliably cause cognitive disruption. If dehydration induced by relatively demanding conditions does not produce reliable changes then it is reasonable to expect that normal daily variations will have a lesser impact. In fact it is difficult to quantify the normal variation in hydration as the fluid intake of free living individuals has been infrequently and poorly recorded. Where it has been recorded it does not begin to reflect the changes, over a day, of complex and dynamic mechanisms. Even these more limited studies have been described as being “in short supply” [51]. 

One of the few studies of the topic examined young adults who were not exercising or living in a hot climate. The mean fluid intake was less than 2.1 L/24 h with a range from 1.4-3.3 L [51]. A similar study considered those aged 40 to 79 years of age living in a temperate climate [52]. Their water intake varied greatly: In men from 1.4 to 7.7 L/day (mean 3.0 L) and from 1.2 to 4.6 L/day in women (mean 2.5 L). Only after 70 years of age was the water intake less than in younger subjects. Thirty-eight percent had a water intake less than the recommended level although this did not result in hyperosmotic dehydration. A survey calculated the fluid intake of over 4000 non-institutionalized Germans over 65 years of age [53] and reported a median intake for men of 1.6 L (5th percentile 0.7 L and 95th percentile 3.0 L). The comparable figures in women were 1.4 L (0.6/2.5 L). These median figures met the German recommendation of an intake of 1.3 L/day but were well below the recommendations of some other countries. Intake did, however, decrease with age. A proportion did not, however, achieve the reference value (33% between 65 and 74 years; 44% between 75 and 84 years; 51% in those over 85 years). As total fluid restriction for 24 h only resulted in a 1.8% decrease in body weight [30], the variation in those going about their everyday life is likely to be much smaller.

It seems that water intake varies greatly among individuals without any obvious sign of problems. In summary the evidence does not allow the conclusion that dehydration is a problem for the cognitive functioning of those living a sedentary life-style. It should, however, be remembered that this conclusion largely reflects a lack of relevant evidence. Simply the hydration status of young adults going about their everyday life has not been related to their cognitive functioning. The speed at which dehydration occurs in temperate conditions again questions the probability that dehydration is going to result from a failure to drink repeatedly during the six to seven hours of a working day. Inevitably fluid will be consumed as part of any food eaten for lunch and in many cases a drink will be consumed at this stage as well as mid-morning and mid-afternoon. 

The possibility remains that vulnerable populations such as older adults may have a higher risk of dehydration induced disruption of cognition. There have even been reports of Dehydration Encephalopathy in older adults, associated with decreased functioning of the frontal lobes and diffuse symptoms such as the appearance of cognitive decline [54]. Some have, however, suggested that age does not necessarily result in physiological changes in the kidney, rather decreased renal functioning reflects various disease states, albeit the incidence of many diseases increase with age [55]. Various age-related alterations in kidney functioning have been described, including “a decrease in kidney size, increased glomerular sclerosis, altered tubular structure, and an altered pattern of vascular flow” [56]. In some individuals renal functioning declines with age due to glomerular sclerosis [57], with an associated decline in the mean glomerular filtration rate. Also the ability to conserve sodium declines and there is a lesser ability to excrete an excess of water as the kidney becomes less responsive to vasopressin [58,59]. These changes make older people potentially susceptible to over-hydration, such that advice concerning increasing hydration should be carefully considered.

Children are another potentially vulnerable group. The four intervention studies that found improved performance in children aged 7 to 9 years [44,45,46,47] offer the only consistent finding in the area of dehydration. These findings can, however, be viewed as surprising as the children studied were likely to be at the most only mildly dehydrated. In one of these studies [47] 58% of the children had drunk both at breakfast and lunch and on 13% of occasions three or more times, although it is possible that only a few sips of drink were consumed. In these studies 200-400 mL were drunk, probably more than would have been consumed if the children had freely consumed until no more was desired. Questions arise about the optimal dose and the ideal pattern of consumption that have not been addressed. 

Unlike the elderly, in children there are very limited data dealing with the homeostatic control of thirst, although again they may differ from young adults as it is suggested that children have immature thirst mechanisms [60]. The possibility exists that children are particularly susceptible to an inadequate fluid intake, given the large surface area to volume, higher levels of activity and immature thirst mechanisms. A study asked 10-12 year olds to cycle at 39 °C, once when they drank when thirsty and on another occasion when forced to drink to replace fluid loss. When only drinking when thirsty the children became progressively dehydrated [61], data consistent with thirst mechanisms not being fully operational at this age. Manz [62] compared the actual and the minimum and maximum urine osmolality to give an indication of the extent to which the capacity to concentrate urine changes with age. The maximum capacity of the kidneys to concentrate urine increases during the first six months of life and during the second and third year of life to reach a level that is characteristic of children, adolescents and young adults. Older adults have a higher minimum and a lower maximum urine osmolality. The median decrease in maximum osmolality, from the level at 20 years of age, was 3.4 mosm/kg/year. Such data suggested that older adults rather than children are at a higher risk of dehydration. It maybe, however, that with children there are social rather than physiological risk factors in that they are not totally in control of their access to fluid. 

If adverse effects of dehydration can in the future be demonstrated then there will be a need to establish the underlying mechanisms. The possibility that hormonal correlates of dehydration may be involved has been suggested [63], including a role for cortisol, vasopressin and prostaglandins. 

In conclusion, in young adults there is little consistent evidence that under everyday conditions dehydration disrupts cognition, although this may simply reflect a general lack of relevant evidence. The possibility that both older adults and children are at a higher risk is a more plausible hypothesis and although again the topic had been little considered the data with children are consistent. 
